# Design and Validation of a Questionnaire to Measure Interprofessional Collaborative Practice for Auditing Integrated Hospital Care: Empirical Research

**DOI:** 10.1097/CEH.0000000000000544

**Published:** 2023-11-21

**Authors:** Mirelle Hanskamp-Sebregts, Petra J. van Gurp, Jozé Braspenning

**Affiliations:** **Dr. Hanskamp-Sebregts:** Radboud University Medical Center, Institute of Quality Assurance and Patient Safety, Nijmegen, The Netherlands. **Prof. van Gurp:** Radboud University Medical Center, Institute of Quality Assurance and Patient Safety, Nijmegen, The Netherlands, and Department of General Internal Medicine, Radboud University Medical Center, Nijmegen, The Netherlands. **Prof. Braspenning:** IQ Healthcare, Radboud Institute of Health Sciences, Nijmegen, The Netherlands.

**Keywords:** interprofessional collaboration, hospital, self-assessment, quality assurance, questionnaire, integrated care pathway

## Abstract

**Introduction:**

Interprofessional teamwork is the key issue of delivering integrated hospital care; however, measuring interprofessional collaboration for auditing is fragmented. In this study, a questionnaire to measure InterProfessional collaborative Practice for Integrated Hospital care (IPPIH) has been developed and validated.

**Methods:**

A four-step iterative process was conducted: (1) literature search to find suitable questionnaires; (2) semistructured stakeholder interviews (individual and in focus groups) to discuss the topics and questions (face validity), (3) pretesting the prototype of the questionnaire in two different integrated care pathways for feasibility, usability, and internal consistency, and (4) testing (content and construct validity and responsiveness) of the revised questionnaire in eight integrated care pathways; the validation and responsiveness was tested by means of exploratory factor analysis, calculation of Cronbach alpha, item analysis, and linear mixed model analysis.

**Results:**

Based on six questionnaires and the opinion of direct stakeholders, the questionnaire IPPIH comprised 27 items. Five different domains could be distinguished: own skills, culture, coordination and collaboration, practical support, and appreciation with the Cronbach alpha varied from 0.91 to 0.48. The self-reported intensity of the collaboration within a specific care pathway significantly influenced the outcome (*P* = .000).

**Discussion:**

The product is a questionnaire, IPPIH, which can measure the degree of interprofessional collaborative practice in integrated hospital care pathways. The IPPIH was initially developed for quality assurance. However, the IPPIH also seems to be suitable as a self-assessment tool for directors to monitor and improve the interprofessional collaboration and the quality of their integrated care pathway.

## Abstract 2

Supplemental Digital Content is Available in the Text.

Interprofessional collaborative practice may improve integrated care and as such health care outcomes.^1^ Integrated hospital care is mostly organized in clinical pathways. In such pathways, care is systematically organized for groups of patients with comparable diseases and on the basis of evidence-based guidelines, protocols, and indicators.^2,3^ An important characteristic of these pathways is the interprofessional collaboration between the different health care providers to deliver the best possible quality of care based on evidence, best practice, and patients' expectations and their characteristics.^2^ Interprofessional collaboration practice allows sharing of expertise and perspectives from different professional backgrounds to jointly define a goal and plan of action together with the patient to achieve the desired outcomes.^4^ The combining of health care providers' resources asks for effective communication, trust, recognition and respect of each other's knowledge, role, and team-agreed responsibilities.^5,6^ Audits as a quality improvement approach can identify areas for improvement and implement changes for the better.^7^ Our research focuses on one of the most important aspects of contemporary auditing (given the increase of patient complexity), namely interprofessional collaboration.^4^ An audit can support a lifelong learning and continuing education on this topic for health care professionals.^8^ However, measuring interprofessional collaborative practice to audit integrated hospital care is still work in progress. A recent study revealed a lot of unclarity between interprofessional education and collaboration in a hospital accreditation program.^9^ Of course, hospital audits make use of instruments that measure teamwork, such as the Team Climate Inventory.^10^ However, this kind of teamwork focuses on health care teams with strong ties.^11^ Integrated care pathways are characterized by less-frequent relationships between team members (loose ties). The composite team often works in different departments and meets occasionally to develop the care plan for a particular patient. However, the interprofessional relationship itself is intense. Interprofessional collaboration relates to person-centered care for example (1) consultation with different health care professionals to exchange information, ideas and recommendations; and (2) develop a shared vision and a focused approach in consultation and with shared responsibility.^12^ This requires different competences, for example the ability to establish and maintain *collaborative* working relationships with different health care providers, the ability to communicate in these relationships effectively in a responsible and responsive manner, and the ability to continually improve the collaborative practice.^13^

Several instruments on measuring interprofessional collaboration are already available.^14,15^ A recent systematic review and a scoping review identified respectively 7 and 29 instruments.^14,15^ However, none of the instruments have been tested in hospital-integrated care pathways. There were also questions on the validation of these instruments because the methodologic quality of the studies was low or moderate.^14^ This may reduce the trustworthiness of the study results. In addition, little was known on the validation for auditing purposes.^14^ It turned out to be impossible to recommend one or a couple of instruments.^14,15^ Commissioned by the audit team of the Radboud University Medical Center, this study aims to overcome the ambiguity of measuring interprofessional education and interprofessional collaborative practice in audit programs. We aim to critically review available instruments with the intent to design and validate a new instrument that can be used for (internal) audits of integrated care pathways. The domains and items will be developed together with the target users, health care providers, and supportive staff of clinical pathways for integrated hospital care. The items from such instruments should be applicable to all clinical pathways in integrated hospital care.

## METHODS

### Study Design and Setting

**FIGURE 1.**
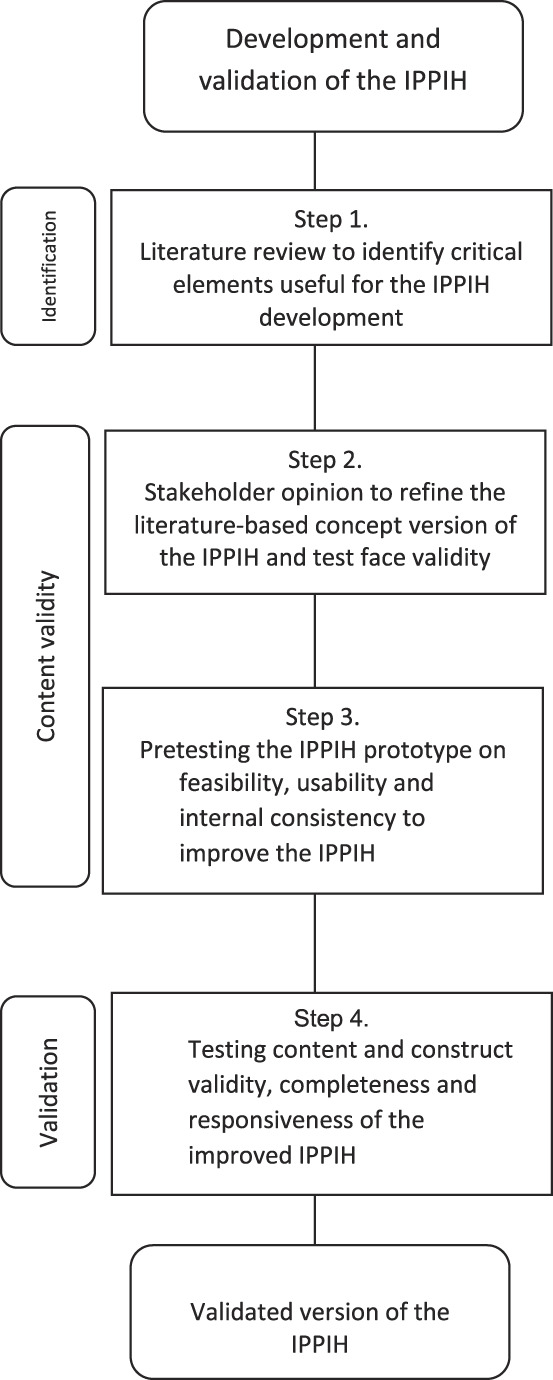
Design of the development and validation of the instrument IPPIH. IPPIH, Interprofessional Collaborative Practice for Integrated Hospital Care

The process of developing and validating the content and construct of the instrument involved four steps (Fig. 1). First, we searched for appropriate domains and/or items in the literature. Second, we used stakeholder opinion to refine the items.^16^ Third, the prototype was tested in two integrated care pathways on feasibility, usability and internal consistency.^16^ Fourth, the improved instrument named “measurement of InterProfessional collaborative Practice for Integrated Hospital care (IPPIH)” was tested and established in eight integrated care pathways.^17,18^ In addition, we studied systematic differences in the characteristics of health care providers and the responsiveness of the new instrument.

The instrument was developed and validated in various oncologic and nononcologic care pathways of the Radboud University Medical Center and partner hospitals in the Netherlands, where a part of the integrated care took place. The development and pretesting were from March 2019 to December 2019, and validation from September 2020 to 2021. The study was approved by the local medical ethical committee of the university medical center (registration number: 2019-5421).

### Literature Review (Step 1)

#### Search Strategy and Databases

To identify appropriate items that reflected the interprofessional collaborative practice, we used a rapid review and assessment of existing instruments on measuring interprofessional collaboration. A rapid review offers a streamlined version of standard systematic review methods in a shorter time frame, with the resultant output a summary.^19^ In March 2019, two researchers (J.B. and M.H.-S.) searched for relevant studies in the period 1970 to 2019 in the following databases PubMed, EMBASE, CINAHL, PsychInfo and Web of Science. The search strategy was repeated in September 2021 for literature updates. The search terms were: interprofessional collaboration, health care providers, teamwork, multidisciplinary teams, patient care, survey, questionnaire, and validity. The search terms were linked together using Boolean logic (AND, OR). In addition, the references of the included studies were manually checked to identify additional relevant studies (snowballing).^20^ J.B. and M.H.-S. independently selected English-language studies that met the following inclusion criteria: (1) the instrument measures team functioning of health care providers from the health care providers' perspective; (2) interprofessional collaboration is a part of care (processes), (3) detailed description of the measuring instrument; (4) reported data on validity; and (5) tested among health care providers.

#### Data Extraction and Methodologic Quality of the Studies

For the data extraction, J.B. and M.H.-S. used a standardized form and registered independently: the purpose of the study, study population, study design and method, results of the reliability, and validity analyses including statistical parameters (see **Appendix**, **Supplemental Digital Content 1**, http://links.lww.com/JCEHP/A280).The methodologic quality of the included studies, on which the instrument was developed, were assessed more broadly using the COSMIN Risk of Bias checklist (see **Appendix**, **Supplemental Digital Content 2**, http://links.lww.com/JCEHP/A281).^17,18^ To determine the overall rating about the methodologic quality of each study, the lowest score of any measurement property was leading.^17^ If there was sufficient methodologic quality, the instrument was included in the selection. The relevant findings were discussed by J.B. and M.H.-S. to define the items that reflected the interprofessional collaborative practice for a literature-based concept. Type of instrument, wording issues, formulation to measure the items, item order, response format, and instrument layout were also discussed by J.B. and M.H.-S. for the instrument design and development.^21^

### Stakeholder Opinion (Step 2)

To refine the literature-based concept of an instrument, we used stakeholders' opinion: four doctors, two nurses, one paramedic, and one senior manager selected on the basis of their expertise in the field of quality assessment of integrated care, and national and local patient representatives (purposive sampling).^22^ These health care providers sent individual written feedback on the relevance, instruction, content, and lay-out of the concept instrument to M.H.-S. M.H.-S. conducted semistructured interviews with the health care providers individually to substantiate and/or clarify the feedback provided (see **Appendix**, **Supplemental Digital Content 3**, http://links.lww.com/JCEHP/A282, for the interview topic guide). Subsequently, two focus groups of health care providers working in different integrated care pathways were held to discuss again the relevance for interprofessional collaboration, the congruence of the selected items, the wording of the items and the instruction (see **Appendix**, **Supplemental Digital Content 3**, http://links.lww.com/JCEHP/A282). The two integrated care pathways differed in interdisciplinary composition and the number of years in function. J.B. was the moderator of the two focus groups. M.H.-S. observed the focus group sessions. Both researchers are experienced interviewers and observers. Field notes were taken during the consultations and focus group sessions. The concept was adapted according to the suggestions given and transformed into a prototype of the instrument.

### Pretesting of the Content Validity (Step 3)

The content validity of the prototype was tested in two integrated care pathways (one oncology and one nononcology). The involved health care providers (*n* = 73), who are medical specialists, nurse practitioners, paramedical staff, and secretarial assistants, were invited (voluntarily) to fill in the IPPIH online within 3 weeks. After 2 weeks, a reminder was sent to the nonrespondents. It was mandatory to fill in all items on professional collaboration and the background information on the health care providers' gender, age, function, number of years working in the pathway, and the intensity of their collaboration by a frequency scale (daily, weekly, monthly, yearly, never).

They were also asked to assess the user-friendliness of the survey, time to fill in, and lay-out. Integrated care pathway directors received their IPPIH results in a feedback report for quality improvement purposes of the integrated care pathway. The feedback report consists of a description of the respondents, tabulated the mean, SD, minimum and maximum of the IPPIH score, and a summary of the main topics in a cobweb chart. One week after receiving, integrated care pathway directors gave their opinions in a dialogue on the quality of the feedback report to M.H.-S. (see **Appendix**, **Supplemental Digital Content 3**, http://links.lww.com/JCEHP/A282, for feedback report topics).

The data of the items that aimed to measure interprofessional collaboration and the respondents' characteristics were statistically analyzed using the statistical program IBM SPSS Statistic 25.0.^23^ Patterns in missing data have been studied by item analysis using descriptive statistics to explain possible lacks of data and to prevent biased results. The internal consistency (Cronbach's alpha) was calculated to determine whether constituent items measure the same construct of interprofessional collaboration in integrated care pathways.^24^ Test results were used to improve the prototype of the instrument, named IPPIH, for validation. **Supplemental Digital Content 4** (see **Appendix**, http://links.lww.com/JCEHP/A283) shows the IPPIH, which is validated.

### Validation of the IPPIH (Step 4)

The procedure for collecting the data for validation of the IPPIH was the same as in the pretest (step 3). The health care providers of eight integrated care pathways (*n* = 182), five oncologic and three nononcologic integrated care pathways, were invited to fill in the online IPPIH. They were asked to complete the IPPIH for a specific integrated care pathway, an oncologic or nononcologic pathway. Some of them work for several pathways, such as the pathologist or radiotherapist. Respondents with more than five missing values were excluded from the statistical analyses. The completeness of the data was checked (item analysis). The background information was analyzed to search for systematic differences in the characteristics of health care providers using a linear mixed model (MLA) analysis with random effects for pathway-level clustering; a significance level of ρ ≤ 0.05 was used. Because health care providers work in integrated care pathways (clusters) within one hospital, an MLA takes clustering of data (scores of the instrument) into account.^24^

An exploratory factor analysis, principal component analysis with Varimax rotation,^24^ was performed to determine the different domains of the instrument. Using principal component analysis also allowed us to remove redundant or unnecessary items of the IPPIH. To structure the proposed factors from the exploratory factor analysis, we used Varimax rotation. Varimax rotation maximized the loadings of items with a strong association with a factor, and minimized those with a weaker factor.^24^ The number of factors of the IPPIH and clustering items together became clearer.

We checked the correlation between the items to assure that every item contributes toward the interprofessional collaboration and have a unique contribution to a factor by accepting a correlation coefficient of <0.70.^24^

Subsequently, the internal consistency was assessed using the Cronbach alpha of the overall IPPIH and per factor. A Cronbach alpha of >0.70 is considered as acceptable.^25^ In addition, the Pearson correlation coefficients (r) were calculated for the correlations between health care providers' and pathway's characteristics; r < 0.40 is considered a low correlation.^24^

To study the responsiveness of the instrument, we explored the effect on the results of the intensity of the collaboration and the type of pathway (oncological or non-oncological) in the MLA analysis.^26^ Integrated care has been practiced longer in oncologic than in nononcologic pathways. We calculated the intraclass correlation coefficient to describe how strongly the different pathways resemble each other; it describes pathway-level clustering by including random effects for each pathway.^27^

## RESULTS

### Literature Review (Step 1)

**FIGURE 2.**
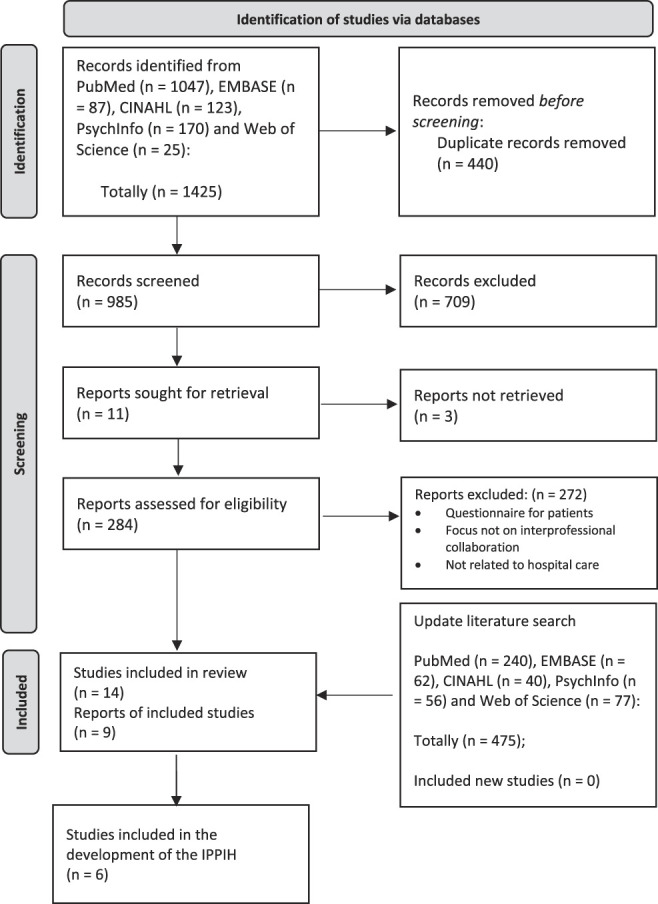
Included studies for developing an instrument to measure interprofessional collaboration in an integrated care pathway

Of the total 1452 studies, 10 studies fully met the inclusion criteria,^13,28–36^ (Fig. 2). Four studies were added because of the explored references.^37–40^ Of the 14 studies, nine were studies^13,30,32–35,37,38,40^ concerned with the development of a questionnaire to measure interprofessional collaboration and one study^39^ consisted of validating indicators for measuring professional collaboration. All instruments contain a theoretical foundation for interprofessional collaboration. The assessed methodologic quality of the studies ranged from inadequate to very good (see **Appendix**, **Supplemental Digital Content 2**, http://links.lww.com/JCEHP/A281). Of the nine questionnaires, none of them seemed to meet the measurement of team functioning in integrated care pathways entirely. The literature study did, however, yield various relevant components. Based on these components and the separate items, a questionnaire was designed based on five development studies^13,35–38^ and the empirical typology from D'Amour's interprofessional collaboration model.^39^ This model identifies four dimensions that characterize the processes of interprofessional collaboration, two related to relationships between individuals (shared goals and vision, and internalization) and two related to the organizational setting (governance and formalization). All these dimensions are interrelated and present in all collective action.^11,29,39^ The repeated search strategy in September 2021 did not yield additional new relevant studies. **Supplemental Digital Content 5** (see **Appendix**, http://links.lww.com/JCEHP/A284) showed the constructs belonging to the items distilled from the six studies. The study characteristics are described in **Supplemental Digital Content 6** (see **Appendix**, http://links.lww.com/JCEHP/A285).

### Development of the IPPIH

The literature-based concept of the IPPIH consisted of a total of 19 items based on the constructs belonging to the items distilled from the six studies^13,35–39^ (see **Appendix**, **Supplemental Digital Content 5**, http://links.lww.com/JCEHP/A284, items 1–19). The items were related to interprofessional collaborative practice. The 19 items were divided into four categories based on content: approach, working atmosphere, organization, and director of the integrated care pathway. These items could be answered using a six-point *agreement* rate: from strongly disagree (1) to strongly agree (5) with a neutral category “neither” (3) and a “not applicable” answer.

### Stakeholders' Opinion (Step 2)

The stakeholders mentioned that the IPPIH is overall a clear and specific survey with relevant items to measure interprofessional collaboration of health care providers in an integrated care pathway. It helps integrated care pathways to measure interprofessional collaborative practice and it can identify areas for improvement. Suggestions have been made on the ordering and rephrasing of some items, and for the instructions. It was also advised to add the answer option “unable to assess” mainly for the health care providers who are less involved in the pathway, such as the pathologist and radiologist. An extra item for the oncologic pathways “Treating “late effects” is explicitly part of our integrated care pathway.” was suggested (patient's perspective) to add to the questionnaire. The concept IPPIH was modified into a prototype according to the given suggestions.

### Pretesting of the Content Validity (Step 3)

In total, 30 of the 73 team members of the two pathways completed the survey, a mean response rate of 41%. The mean score on the IPPIH was 4.22 (SD = 0.72). The time to fill in the IPPIH was approximately 10 minutes. Respondents indicated that the short questionnaire was pleasant to fill in and that the items about interprofessional collaboration were clear.

It was suggested to change the item on the frequency of the collaboration (“how often do the providers meet each other”) into an item on the intensity of the relationship in the integrated care pathway; rating on a scale 1–10 (1 = minimal collaboration; 10 = maximal collaboration), as it reflected better the degree of interaction. Furthermore, it was recommended to use a seven-point *quality* rating scale, ranging from excellent (6) to poor (1) and a “cannot assess” answer. Finally, it was proposed to add information on the interprofessional competence of the individual members. Eight items of the validated Interprofessional Collaborative Competencies Attainment Survey^13^ were incorporated in the IPPIH to measure individual competences (see **Appendix**, **Supplemental Digital Content 5**, http://links.lww.com/JCEHP/A284, items 20–27). **Supplemental Digital Content 4** (see **Appendix**, http://links.lww.com/JCEHP/A283) presents the items of the prototype after pretesting. Subsequently, this prototype of the IPPIH, has been validated.

### Validation of the IPPIH (Step 4)

#### Respondent and Response Characteristics

Total 119 of the 182 participants (65.4%) in the eight integrated care pathways responded (Table 1). Most of the respondents were working in an oncologic integrated care pathway (70.6%) and were clinicians (77.3%). They worked minimum 1 year and maximum 40 years in the integrated care pathway with a mean of 8.1 years (SD 7.7). The age of the respondents varied from 28 to 66 years with a mean of 46.7 years (SD 9.6).

**TABLE 1.** Respondent and Response Characteristics

Characteristics	Response% (*n*)
Respondents/total invited health care providers	65.4 (119/182)
Integrated care pathway	
Oncology	70.6 (84/119)
Nononcology	29.4 (35/119)
Gender	
Male	41.2 (49/119)
Female	58.8 (70/119)
Function	
Medical	77.3 (92/118)
Nursing	16.8 (20/118)
Paramedical	1.7 (2/118)
Others	3.4 (4/118)
	Mean (SD) Min–Max
Age (y) (*n* = 113)	46.7 (9.6) 28–66
Working in integrated care pathway (y) (*n* = 107)	8.1 (7.7) 1–40

The number of respondents varied per respondent characteristic because of incorrect data.

The MLA indicated that none of the health care provider's characteristics affected the IPPIH score significantly (Table 2).

**TABLE 2.** The Impact of Background Characteristics on the IPPIH Score

Variables	Interprofessional Collaborative Practice for Integrated Hospital Care (*n* = 107*)					
	Model 0			Model 1		
	*Ь* (SE_*Ь*_)	95% CI	*P*	*Ь* (SE_*Ь*_)	95% CI	*P*
Constant	0.311 (0.043)	NA	.000*	2.961 (0.563)	1.841 to 4.080	.000†
Gender‡				0.040 (0.112)	−0.183 to 0.264	.718
Age				−0.001 (0.001)	−0.004 to 0.000	.199
Function group§						
Nurse				0.328 (0.533)	−0.730 to 1387	.540
Manager				0.535 (0.738)	−0.930 to 2001	.470
Director				0.621 (0.553)	−0.477 to 1720	.264
Paramedic				0.587 (0.742)	−0.886 to 2061	.431
Others				0.594 (0.521)	−0.441 to 1631	.257
Working in the pathway (y)				−0.009 (0.006)	−0.021 to 0.002	.133
Intensity of collaboration				0.126 (0.027)	0.071 to 0.181	**.000** †
Type of pathway‖				0.171 (0.208)	−0.322 to 0.664	.439
ICC	0.186 (18.6%)					

* Three respondents filled in an undetectable characteristic.

† *P* ≤ .05. Significant *P* values are in bold.

‡ Male = 0.

§ Clinicians as reference group.

║ Nononcology integrated care pathways = 0.

CI, confidence interval; ICC, intraclass correlation coefficient; IPPIH, interprofessional collaborative practice for integrated hospital care; NA, not applicable; SE, standard error.

### Responsiveness

**FIGURE 3.**
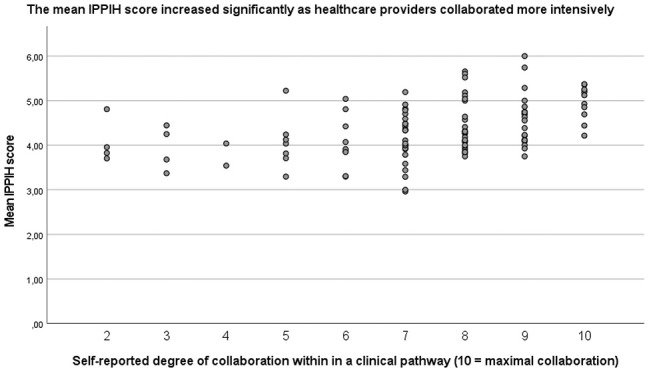
The more intensive health care providers work together, the higher the mean and less variability of the IPPIH score. IPPIH, Interprofessional Collaborative Practice for Integrated Hospital Care

Health care providers within a nononcological pathway worked more intensive together than the health care providers within an oncologic pathway, respectively 7.8 and 6.9 on the intensity scale of 1–10. There is a significant correlation between the intensity of interprofessional collaboration and the mean IPPIH score (Table 2). Respondents with a higher intensity score had a higher mean and less variability of the IPPIH score (Fig. 3).

### Item Analysis

Nine of the 119 respondents gave the answer “cannot assess” on more than five items. These respondents were for that reason excluded for the exploratory factor analysis and reliability analysis (internal consistency). Additional analysis showed that these respondents were unable to assess some items (eg, a resident who recently started working in the integrated care pathway). This concerned, for example, the items: establishing the care and treatment plan together with the patient (and/or their informal caregivers) (item 27) and adequately support the integrated care pathway in conducting the care tasks (item 16). The items with the highest mean scores on the IPPIH were items: 1 (approach each other easily), 3 (confidence in the expertise of my colleagues in the integrated care pathway), and 18 (the director of the integrated care pathway is open to ideas and concerns from integrated care pathway participants), see **Supplemental Digital Content 7** (see **Appendix**, http://links.lww.com/JCEHP/A286). The items with the lowest mean score were: 10 (to pay attention to each other's personal well-being), 16 (actively support innovation in the supply of the integrated care pathway), and 20 (promoting effective communication between colleagues in the integrated care pathway). Items with the highest “cannot assess” score were: 9, 16, and 27. Items 15 and 20 (comparable high loadings on two factors) were assigned to the factors that best fit the description of the construct (factor).

### Exploratory Factor Analysis and Reliability Analysis

The extra item about the late effects was excluded in this validation because this item only applies to the oncologic care pathways. Five factors were drawn by exploratory factor analysis (see **Appendix**, **Supplemental Digital Content 7**, http://links.lww.com/JCEHP/A286). The factors jointly explained 66.7% of the variance in the responses. The five factors were related to: (1) “own skills” (*n* = 8), (2) “culture” (*n* = 8), (3) “coordination and collaboration” (*n* = 6), (4) “practical support” (*n* = 3) and (5) “appreciation” (*n* = 2). The internal consistency of the total IPPIH is 0.953. The internal consistency (Cronbach alpha) per factor was respectively: 0.91 (factor 1), 0.89 (factor 2), 0.87 (factor 3), 0.62 (factor 4), and 0.48 (factor 5), which is good for factors 1 to 3, questionable for factor 4 and factor 5 has a poor internal consistency. Three correlations exceeded just >0.70; however, the contents of these items measure different aspects (see **Appendix**, **Supplemental Digital Content 8**, http://links.lww.com/JCEHP/A287). Based on the results of the factor analysis and reliability analysis, all items (*n* = 27) were kept in the first validated version of the IPPIH.

### Feedback Report

The feedback report successively consisted of: a short description of the measurement period and respondent's characteristics, a frequency table of the intensity scores, representing the mean IPPIH scores graphically by a cobweb chart with the three highest and three lowest scores circled, and tabulation of the items (mean, SD, and minimum and maximum IPPIH scores). The integrated care pathway directors found this way of reporting clear and gave no suggestions for quality improvement.

## DISCUSSION

### Principal Findings

The IPPIH measures five factors of interprofessional collaboration within an integrated care pathway: “own skills,” “culture,” “coordination and collaboration,” “practical support” and “appreciation.” The internal consistency of the IPPIH is acceptable. The intensity of interprofessional interaction affected significantly the mean IPPIH score, which means that the instrument is responsive for its purpose. Gender, age, function, the number of years working in the integrated care pathway, and type of the integrated care pathway did not affect the mean IPPIH score. This background information is not necessary to interpret the IPPIH score and thus does not need to be collected for auditing purposes, which helps to maintain the anonymity of the respondent. Stakeholders indicated that the IPPIH is able to measure the interprofessional collaboration of health care providers within an integrated care pathway.

### Strengths and Weaknesses

A strength of this study is the development of the instrument together with the stakeholders. Stakeholder involvement is a known powerful instrument in the feasibility and acceptance of innovations and measures of quality of care.^41,42^ To further support this argument, we can state that the IPPIH, which was initially developed for quality assurance, is now voluntarily used in integrated care pathways in the Radboud University Medical Center as a self-assessment tool to monitor and improve the interprofessional collaboration and the quality of their integrated care. A limitation of the study may be that we developed and tested the instrument in one university medical center. For the generalizability of the study findings, future research should include a larger sample of integrated care pathways and from different hospitals. However, the developed IPPIH was not restricted to one integrated pathway, but eight different pathways could be included. Another limitation may be the response rate. On average, 65.4% of the respondents filled in the web-based questionnaire. Perhaps this is what can be expected in a hospital setting. For instance, Cunningham et al found an overall survey response rate among physician specialists (most of our sample) of 35.0%.^43^

### Validation

According to the internationally highly regarded COSMIN Framework,^17^ we can conclude that we developed an appropriate instrument to measure interprofessional collaboration for auditing integrated health care pathways in a hospital setting. The internal consistency for the factors “own skills,” “culture” and “coordination and collaboration” were high, and acceptable for the other two factors mainly because they consisted of just three and two items. The item about active support of innovation in the pathway could be assigned to several factors. Perhaps this has to do with the innovation that comes to mind. According to the narratives of the factors, this item was assigned to factor 4 “practical support.”

Substantially, nonresponse of some items could be explained by the function of the respondents. In some integrated care pathways, the supportive administrative staff was invited to fill in the questionnaire as well. We relied on the information of the director of the pathway to invite all involved to participate. They informed us that for teambuilding, it was very important to invite this function group as well. It was more important that their voices were heard than that they could each report on every item.

### Constructs of the Instrument in the Context of International Literature

The construct “own skills” was added to the instrument by a stakeholder in one of the integrated care pathways in the pretesting (step 3). This is a strong and congruent factor that is often used in questionnaires that measure interprofessional education such as the Interprofessional Collaborative Competencies Attainment Survey.^13^ These items are introduced by our stakeholders because they represent the necessary competences needed to perform interprofessional collaboration. This addition ties in well with the discussion about ambiguity between education and performance in accreditation programmes.^9^ Our study shows the stakeholders' value to include this component.

Culture plays an important role in many teambuilding instruments such as the Team Climate Inventory.^10^ An open and safe culture matters to achieve cooperation and therefore also interprofessional cooperation.^44^ Participative safety, all team members feel able to propose new ideas and problem solutions in a nonjudgemental climate, is an import factor for optimal collaboration between health care professionals who offer integrated care.^36^

The items belonging to “coordination and collaboration” load high on this construct. These items get to the heart of the instrument, because they address the actual interprofessional collaborative practice. These items are as strongly correlated in the IPPIH as similar instruments with coordination or collaboration as factors for interprofessional care practice such as the Interprofessional Collaborative Competencies Attainment Survey,^13^ ITEM,^40^ and the AICTS.^32^

Interprofessional collaborative practice needs practical support as well. Appointing a dedicated director of an integrated care pathway is necessary support for the development and maintaining of interprofessional collaboration;^39,40^ however, that was already realized in our hospital setting. However, it was noticed that ICT and capacity should be aligned to the interprofessional collaborative practice as well. These are necessary preconditions, just as the management itself. Maybe two items on this subject are too few, but with these items, at least the topic can be addressed. Lemieux-Charles and McGuire^40^ demonstrated in their model (ITEM), the positive influence of resources on health care team effectiveness.

The last construct is about appreciation. That seems like a good lubricant for the interprofessional collaborative practice. The appreciation can come from the patients and from the colleagues. Using outcome measures, such as patient's satisfaction and perceived task outcomes and well-being by team members is important for monitoring the care process and team effectiveness in integrated care.^28,40^

### Scientific and Practical Implications

To confirm the robustness of this result, we suggest to repeat the analysis in other hospital auditing programs.^16^ A confirmative factor analysis can be used to question the classification of our items.^24^ Furthermore, it would be interesting to link the IPPIH-score to quality of care outcomes, for example adverse events or length of stay, to demonstrate a relationship between interprofessional collaboration in integrated care pathways and better patient care. This makes it more urgent to implement interventions to improve or intensify the interprofessional collaboration of health care providers in an integrated care pathway.

For directors of integrated care pathways, who want to start setting up or redesigning an integrated care pathway, the IPPIH provides insight into the important elements of interprofessional collaboration in integrated care. When more IPPIH data become available, reference figures can be calculated for specific pathways. This concrete information can be an extra incentive for improvements. The IPPH is used as standard in our hospital audits to measure periodically, the interprofessional collaboration of certain individual integrated care pathways.

## CONCLUSIONS

The product of this research is a questionnaire for (internal) auditing with good content validity to assess the degree of interprofessional collaboration between health care providers working in an integrated care pathway.

**Lessons for Practice**

■ The IPPIH, which was initially developed for quality assurance, also seems to be suitable as a self-assessment tool to monitor and improve the interprofessional collaboration and the quality of an integrated care pathway.
■ For directors of integrated care pathways, who want to start setting up or redesigning an integrated care pathway, the IPPIH provides insight into the important elements of interprofessional collaboration in integrated care.
■ The intensity of interprofessional interaction affected the mean IPPIH score significantly, which makes it more urgent to implement interventions to improve or intensify the interprofessional collaboration of health care providers in an integrated care pathway.
